# Impact of co-fermentation with *Debaryomyces hansenii* and *Bacillus subtilis* on the flavor characteristic of dark tea infusion

**DOI:** 10.3389/fnut.2026.1806223

**Published:** 2026-04-09

**Authors:** Jin-Rong Ma, Lu-Lu Liu, Jiang-Miao Zhang, Yuan-Liang Wang, Ai-Xiang Hou, Lin Hu, Ming-Zhi Zhu, Yu-Lian Chen, Yu Xiao, Wen-Ping Kong

**Affiliations:** 1 College of Food Science and Technology, Hunan Agricultural University, Changsha, China; 2 Key Laboratory of Ministry of Education for Tea Science, College of Horticulture, Hunan Agricultural University, Changsha, China; 3 Institute of Cotton and Sericulture, Hunan Academy of Agricultural Sciences, Changsha, China; 4 School of Nursing, Gannan Medical University, Ganzhou, China

**Keywords:** *Bacillus subtilis*, co-fermentation, *Debaryomyces hansenii*, flavor characteristic, molecular docking, volatile organic compounds

## Abstract

*Debaryomyces* and *Bacillus* are dominant microbial groups in dark teas such as Fu brick tea and play a key role in the formation of their characteristic flavors. In this study, dark tea infusion was co-fermented using *Debaryomyces hansenii* and *Bacillus subtilis* isolated from Fu brick tea. A comprehensive analytical approach was employed, including headspace solid-phase microextraction coupled with gas chromatography–mass spectrometry (HS-SPME-GC-MS), high-performance liquid chromatography (HPLC), relative odor activity value (rOAV) analysis, molecular docking, and sensory evaluation, to systematically investigate changes in tea polyphenols, catechins profile, and volatile organic compounds (VOCs) during fermentation and to identify key aroma-active substances. The results demonstrated that co-fermentation significantly reduced the contents of total tea polyphenols (by 45.12%) and ester catechins (by 92.63%), while promoting the accumulation of gallic acid. Volatile component analysis revealed that the co-fermentation integrated the aromatic profiles of both monocultures, retaining key aroma compounds such as linalool and nonanal, and introducing unique constituents such as α-cadinol, collectively forming a complex aroma profile dominated by floral, fruity, and woody notes. Molecular docking analysis confirmed strong binding affinity between key floral aroma compounds (e.g., linalool) and the olfactory receptor OR1A1. Sensory evaluation indicated that the co-fermented tea infusion achieved the highest overall scores in aroma and taste, exhibiting a mellow and sweet aftertaste along with a pure, bright, and refreshing aroma, highlighting the synergistic effect of co-culture fermentation. This study reveals that co-fermentation with *D. hansenii* and *B. subtilis* can effectively improve the flavor quality of dark tea infusion through synergistic regulation of polyphenol transformation and volatile compounds formation, providing a controllable bioprocess for the development of flavor-oriented, quality-stable fermented tea beverages with potential for industrial scale-up and commercial application.

## 1 Introduction

Dark tea, a unique post-fermented tea originating in China, is widely favored by consumers for its distinctive flavor profile and health benefits (1–4). Recent advances have begun to decode the complex odor-active compounds that underpin its characteristic aroma, revealing their formation pathways and sensory contributions (5). This flavor complexity is primarily attributed to the intricate metabolic activities of microorganisms during fermentation (6–8). By secreting various extracellular enzymes, these microorganisms systematically transform macromolecules in tea leaves, such as polyphenols, polysaccharides, and proteins, thereby establishing the material foundation for the mellow taste and unique aroma of dark tea (3, 9, 10).

Traditional dark tea is defined by its microbial solid-state fermentation, with fungi (such as *Aspergillus, Eurotium*, and *Debaryomyces*) and bacteria (including *Bacillus* and *Pseudomonas*) identified as crucial functional microorganisms and key contributors to the development of its characteristic quality (11, 12). However, spontaneous solid-state fermentation relies on complex and unstable microbial communities, leading to inconsistent product quality and flavor profiles, which hinders industrial standardization. Therefore, isolating key functional strains and establishing controllable, efficient pure-culture or co-culture fermentation systems are of great significance for elucidating flavor formation mechanisms, achieving targeted flavor modulation, and developing tea beverages with stable quality. In recent years, numerous studies have elucidated the role of individual microorganisms in the formation of tea quality during solid-state fermentation. For instance, solid-state fermentation with *Eurotium cristatum* significantly influences the flavor profile of dark tea, leading to a notable increase in flavor compounds such as (*E, E*)-2,4-heptadienal, linalool oxides, methyl salicylate, methyl 2-methylbutyrate, and ethyl acetate (3). Li et al. (13) reported that fermentation with *Aspergillus niger* improved that aroma and taste characteristics of dark tea. Ma et al. (14) showed that the *Aspergillus luchuensis* P1 (isolated from pu-erh tea) significantly alter phenolic compound profiles via diverse catalytic reactions, thereby playing a crucial role in shaping tea quality during fermentation. It is particularly noteworthy that *Debaryomyces* and *Bacillus* are both dominant microbial genera in dark teas such as Fu brick tea, and their regulatory roles in solid-state fermentation have been preliminarily elucidated. Our previous research found that solid-state fermentation with *Debaryomyces hansenii* upregulates genes related to terpenoid synthesis pathways, promoting the production of floral and minty aroma compounds such as phenylethyl alcohol and linalool (15), while also increasing theabrownins content, resulting in a fuller-bodied tea infusion (16). On the other hand, solid-state fermentation with *Bacillus subtilis* isolated from Fu brick tea effectively degrades bitter and astringent esterified catechins and promotes the substantial generation of floral, fruity, and woody aroma compounds such as linalool, β-ionone, and cedrol (17). Although extensive research has identified over 1,000 volatile organic compounds (VOCs) in FBT and elucidated their formation pathways (4), these studies have primarily focused on traditional solid-state fermentation systems. However, the functional roles of key dark tea microorganisms, such as *D. hansenii* and *B. subtilis*, in liquid-state fermentation—particularly their synergistic effects during co-fermentation—remain largely unexplored. This knowledge gap limits our understanding of their collaborative metabolic potential and hinders the development of controlled fermentation strategies for flavor optimization.

To precisely investigate the synergistic interaction between *D. hansenii* and *B. subtilis* and to develop a more controllable process, this study employed a liquid-state fermentation system. Liquid-state fermentation, as a modern fermentation mode with high controllability, short cycles, and easy standardization, provides an effective approach for precisely regulating tea infusion flavor (18, 19). Liquid fermentation with *E. cristatum* significantly increases floral components such as linalool while degrading esterified catechins, shifting the tea infusion flavor from astringent to floral and fruity (20). *Aspergillus tubingensis* can transform flavonoid glycosides through its specific glycoside hydrolases, shaping the herbal and woody notes of dark tea during liquid fermentation (21). Studies have also shown that liquid fermentation of tea infusion using microorganisms such as *A. niger* and *Aspergillus oryzae* can significantly alter its volatile composition, thereby directionally shaping aroma characteristics (22, 23). Liquid fermentation by *A. niger* has been shown to accumulate linalool and its oxides, α-terpineol, and other compounds, imparting fungal and woody characteristics to the tea (19). In recent years, numerous studies have demonstrated that co-culture fermentation often leverages synergistic or symbiotic relationships among microorganisms, demonstrating greater potential in flavor complexity and quality balance, compared to single-strain fermentation (24). For example, co-fermentation involving *Monascus* with yeast (25), or *E. cristatum* with *A. niger* (19, 26), has proven superior to mono-culture treatments in enhancing aroma richness and improving the color and taste of dark tea. Similarly, multi-strain collaboration can more effectively promote the conversion of esterified catechins, accumulation of free amino acids, and increased diversity of volatile compounds, thereby improving the quality of lower-grade raw tea materials (27–29). These studies collectively suggest that constructing a rational co-culture fermentation system is an effective strategy for fully exploiting microbial metabolic potential and optimizing the flavor quality of fermented tea.

Thus, this study firstly introduces a co-fermentation approach utilizing *D. hansenii* and *B. subtilis* in raw dark tea infusion. By integrating multiple analytical techniques, including high-performance liquid chromatography (HPLC), headspace solid-phase microextraction–gas chromatography–mass spectrometry (HS-SPME-GC-MS), relative odor activity value (rOAV) analysis, molecular docking, and sensory evaluation, this research systematically analyzes the impact of co-fermentation on the metabolic transformation of polyphenolic compounds (particularly ester catechins) and its regulatory effect on the composition of VOCs and key aroma-active substances. Furthermore, the perception mechanism of key aroma compounds was explored by molecular docking analysis. This study aims to elucidate the flavor formation principles and synergistic effects of co-fermentation with *D. hansenii* and *B. subtilis*, thereby providing a technical approach for developing flavor-oriented, quality-stable fermented tea beverages.

## 2 Materials and methods

### 2.1 . Materials and reagents

*D. hansenii* and *B. subtilis* strains were previously isolated from Fu brick tea and are maintained in the Food Microbiology Laboratory culture collection at Hunan Agricultural University (15, 17). Raw dark tea was provided by Hunan Jiuyang Tea Co., Ltd. Acetonitrile and formic acid (chromatographic grade) were purchased from Shanghai Macklin Biochemical Technology Co., Ltd. The C7-C40 n-alkane standard mixture and ethyl decanoate (99.99%) were sourced from Sigma-Aldrich (USA). Sodium chloride was obtained from Sinopharm Chemical Reagent Co., Ltd. (Shanghai, China). Gallic acid and catechins standards (purity ≥ 98%) were purchased from Shanghai Aladdin Biochemical Technology Co., Ltd.

### 2.2 . Preparation of *D. hansenii* and *B. subtilis* starter cultures

The inoculum of *D. hansenii* was prepared by initially reviving the strain in YPD broth under shaking at 120 rpm and 28°C for 48 h. Yeast cells were harvested, washed twice, and resuspended in sterile physiological saline. Cell density was determined using a hemocytometer and adjusted to 10^8^ cells/mL. For *B. subtilis*, the inoculum was developed by first culturing the bacterium in 50 mL of lysogeny broth (LB) at 37°C for 24 h. A 3 mL aliquot of this primary culture was transferred into 150 mL of fresh LB medium and incubated at 37°C for 12 h. Bacterial cells were collected by centrifugation at 8,000 × g for 5 min at 4°C, washed twice with sterile saline, and finally resuspended in sterile physiological saline to a final concentration of 10^8^ cells/mL.

### 2.3 Preparation of co-fermented dark tea infusion using *D. hansenii* and *B. subtilis*

The dark tea infusion was prepared based on a previously established method in our laboratory with minor modifications (15). Briefly, 20.0 g of raw dark tea was precisely weighed into a 2 L erlenmeyer flask. Distilled water was added at a solid-to-liquid ratio of 1:60 (g:mL), resulting in a total volume of 1,200 mL. The mixture was subjected to extraction in a 100°C water bath for 30 min. After extraction, the infusion was filtered through sterile cheesecloth to remove tea residues. The filtrate was readjusted to 1,200 mL with distilled water. The tea infusion was then dispensed into 250 mL erlenmeyer flasks (100 mL per flask) and was sterilized by autoclaving at 121°C for 20 min. The sterilized infusion was allowed to cool to room temperature prior to inoculation.

For fermentation, the prepared *D. hansenii* or *B. subtilis* cell suspensions were inoculated separately into the tea infusion at an inoculum size of 2% (v/v) to prepare single-strain fermented samples, designated as DHF (*D. hansenii* fermented) and BSF (*B. subtilis* fermented). To prepare the co-fermented sample (BDF), the two cell suspensions were mixed at a volume ratio of 1:3 (v/v), and the mixed suspension was inoculated into the tea infusion at a total inoculum size of 2% (v/v). A non-fermented control (NF) was prepared by adding an equivalent volume (2%, v/v) of sterile distilled water to the tea infusion, followed by the same fermentation process. All inoculated infusions were incubated in a constant temperature incubator at 34°C for 8 days. After fermentation, the samples were centrifuged at 4°C and 8,000 × g for 10 min. The resulting supernatants, representing the final fermented dark tea infusions, were collected. All samples were aliquoted and immediately frozen at −20°C for subsequent analyses.

### 2.4 Sensory evaluation

Sensory evaluation was performed according to the Chinese National Standard GB/T 23776-2018 (Methodology for Sensory Evaluation of Tea) by a panel of eight trained assessors (four males and four females, aged 25–45 years) from the Tea Science Department of Hunan Agricultural University, all of whom had completed a systematic one-week training program. Each sample (40 mL infusion) was presented in a blind-coded, randomized order for the assessment of aroma and taste. The aroma was evaluated after transferring the infusion to a sealed 50 mL centrifuge tube, gently shaking, and immediately smelling to minimize volatile loss; and the taste was assessed by taking small sips to ensure full oral coverage. To avoid carryover effects, assessors rinsed with water and waited 1 min between samples. Quantitative scoring was applied to each attribute using defined criteria. Aroma was categorized into three levels: “Pure and Refreshing” (8–10 points), “Relatively Pure, Moderately Strong, and Free of Off-odors” (5–7 points), and “Presence of Off-odors” (1–4 points). Taste was similarly classified into: “Mellow with Sweet Aftertaste” (8–10 points), “Moderately Mellow, Slightly Bitter/Astringent, and No Sweet Aftertaste” (5–7 points), and “Strongly Bitter/Astringent” (1–4 points). The final sensory score for each sample was calculated as the mean of all panelists' ratings.

### 2.5 Determination of total polyphenols content

The total polyphenols content (TPC) in tea infusion samples was determined using the Folin-Ciocalteu colorimetric method (30). A standard curve was first established using a series of gallic acid solutions (0–200 μg/mL). The regression equation derived was y = 0.0027x + 0.0096 (R^2^ = 0.9858). Under consistent analytical conditions, the absorbance values of all dark tea infusion samples were recorded. The TPC was subsequently determined by interpolation from the established calibration curve. Data are reported as mg of gallic acid equivalents per g of sample (mg GAE/g).

### 2.6 Determination of catechins and gallic acid content

Based on a previously described method (31), the concentrations of gallic acid and eight key catechins—catechin (C), epigallocatechin (EGC), gallocatechin (GC), epicatechin gallate (ECG), epicatechin (EC), gallocatechin gallate (GCG), epigallocatechin gallate (EGCG), and catechin gallate (CG)—were measured using high-performance liquid chromatography (HPLC). Analysis was performed on a C18 column maintained at 30°C with a mobile phase consisting of (A) 0.1% formic acid in ultrapure water and (B) acetonitrile. The gradient program was set as follows: 10–35% B over 0–40 min, then returning to 10% B from 40 to 42 min. The flow rate was 0.8 mL/min, and detection was conducted at 280 nm. Identification and quantification of individual compounds were accomplished by comparing retention times and UV spectra with those of certified reference standards.

### 2.7 Analysis of VOCs

VOCs in fermented dark tea infusions were collected using headspace solid-phase microextraction (HS-SPME). Specifically, 6 mL of dark tea infusion was placed into a 20 mL headspace vial along with 0.5 g of NaCl and 10 μL of ethyl decanoate (internal standard). Ethyl decanoate was selected as the internal standard due to its chemical stability, absence in the native tea matrix, and a retention time that does not interfere with the peaks of the target volatile compounds in dark tea infusion. The vial was sealed with a polydimethylsiloxane septum. During extraction, the sample was equilibrated at 80°C for 10 min with continuous stirring at 600 rpm. A 50/30 μm DVB/CAR/PDMS SPME fiber was then exposed to the headspace for 50 min to adsorb VOCs. Following adsorption, the fiber was thermally desorbed in the GC injector port at 240°C for 5 min in splitless mode. VOC separation was performed on an HP-5MS capillary column (30 m × 0.25 mm × 0.25 μm; Agilent, USA) using an Agilent 7000D GC–MS system. The GC oven temperature program was as follows: initial temperature 40°C (held for 3 min); increased to 80°C at 2°C min^−1^ (held for 2 min); ramped to 150°C at 3°C min^−1^; then to 180°C at 5°C min^−1^; finally to 230°C at 15°C min^−1^ (held for 2 min). Mass spectrometry detection was performed in electron ionization (EI) mode at 70 eV, with an ion source temperature of 230°C and a scanning range of m/z 35–400.

Retention indices (RIs) were established by injecting a C7–C40 n-alkane standard mixture under identical GC–MS conditions. Putative identification of volatile organic compounds (VOCs) was based on matching experimental mass spectra and calculated RIs with reference data in the NIST 17 library. Given the complexity of the dark tea matrix and the difficulty of acquiring pure standards for all detected compounds, a semi-quantitative method was employed. The peak area of each VOC was normalized against that of the internal standard (ethyl decanoate, 8.64 mg L^−1^), and its relative concentration was derived accordingly. To assess the potential contribution of each VOC to the overall aroma, the relative odor activity value (rOAV) was calculated as rOAV = C_*i*_/ OT_*i*_, where C_*i*_ is the relative concentration of the compound and OT_*i*_ represents its odor threshold in water (31, 32).

### 2.8 Molecular docking study of VOCs interactions with olfactory receptors

Following the method reported by Liang et al. (33), three-dimensional (3D) structural models of the VOCs were retrieved from the PubChem database (https://pubchem.ncbi.nlm.nih.gov). The 3D structures of the olfactory receptors were obtained from the UniProt database (https://www.uniprot.org). Molecular docking was performed using CB-Dock2, an automated tool for cavity detection and docking. The grid box was automatically defined to encompass the predicted binding pockets of the receptors. The exhaustiveness level was set to the default value 8 to ensure a balance between accuracy and computational efficiency. Docking poses with the highest predicted binding affinity (lowest binding energy) were selected for further analysis. Subsequently, BIOVIA Discovery Studio 2019 software was performed to visualize and analyze the binding interactions in both 3D and 2D formats.

### 2.9 Statistical analysis

All experimental procedures were conducted in triplicate. Statistical analysis of the data was performed with SPSS 26, while graphical representations were prepared using Origin 2018. Multivariate statistical analysis was conducted using the online platform Metware Cloud (https://cloud.metware.cn). Heatmaps were created using TBtools software.

## 3 Results and discussion

### 3.1 . Effects of co-culture fermentation on polyphenols and catechins in dark tea infusion

**Figure 1:**
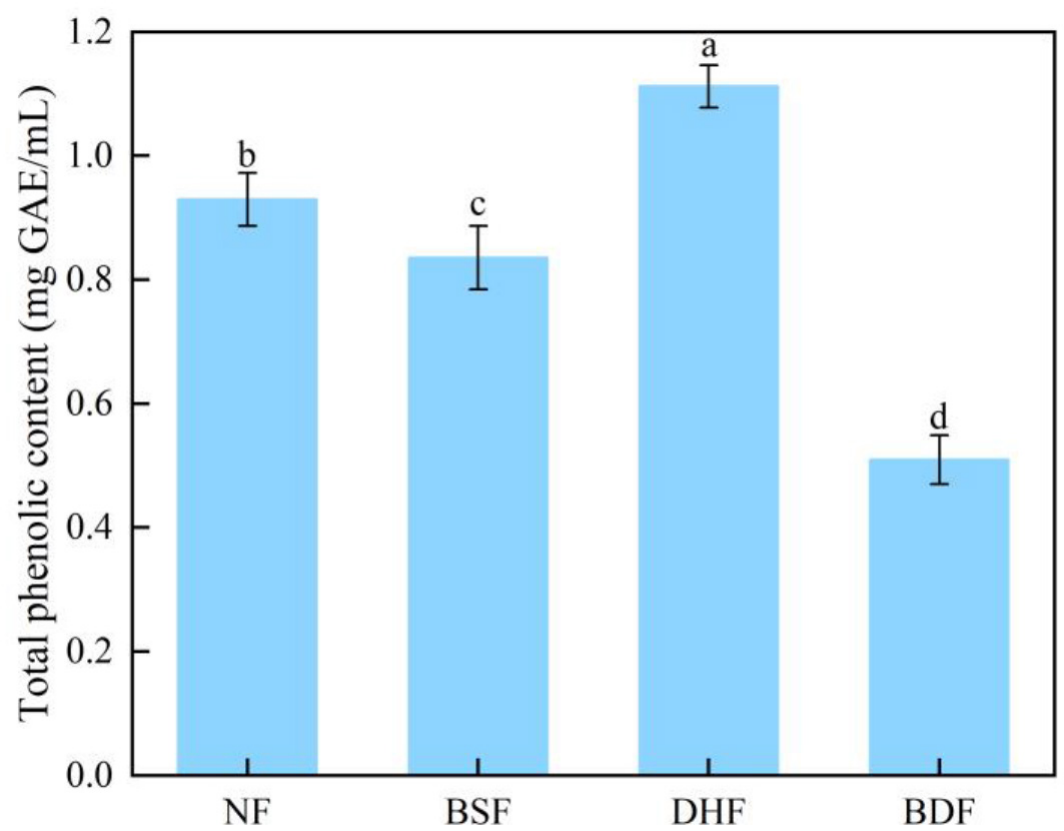
Differences in total polyphenols content among different dark tea infusions. Means followed by different letters indicate statistically significant differences (*p* < 0.05) among the samples. NF, non-fermented control; BSF, *B. subtilis* mono-culture fermented; DHF, *D. hansenii* mono-culture fermented; BDF, co-fermented with *D. hansenii* and *B. subtilis*.

Tea polyphenols constitute the primary components responsible for the bitter and astringent taste of tea (34). As shown in Figure 1, compared to the unfermented control, mono-culture fermentation with *D. hansenii* significantly increased the total polyphenols content in the tea infusion by 19.35% (*p* < 0.05). This phenomenon may be attributed to the hydrolysis of bound phenolic compounds (e.g., phenolic acid esters, flavonoid glycosides) in tea leaves by hydrolytic enzymes, such as β-glucosidase and esterase, secreted by the yeast, thereby releasing more free phenolic forms (35). Additionally, the sufficient hydrolysis of cell wall polysaccharides by microbial enzymes (e.g., cellulase, pectinase), particularly the disruption of linkages within the polysaccharide-lignin network, could lead to the release of more polyphenols (36). In contrast, mono-culture fermentation with *B. subtilis* decreased the total polyphenols content by 9.68%. This bacterial strain typically secretes extracellular oxidases, including polyphenol oxidase and peroxidase, which can catalyze the oxidation, polymerization, and even degradation of phenolic compounds, leading to a net consumption of total polyphenols (37). Notably, co-culture fermentation with *D. hansenii* and *B. subtilis* exhibited a significant synergistic effect. Compared to the unfermented control and the mono-culture fermentations with *B. subtilis* and *D. hansenii*, the total polyphenols content in the co-culture fermented tea infusion was significantly reduced by 45.12%, 38.96%, and 54.14%, respectively. This effectively reduced the potential sources of bitterness and astringency. Consistent with our findings, fermentation of summer-autumn green tea with *Eurotium cristatum* significantly decreased tea polyphenols and alleviated bitter and astringent tastes (38). We hypothesize that this synergistic effect arises from a sequential two-step metabolic pathway: the hydrolytic enzymes secreted by the yeast first cleave high-molecular-weight or bound polyphenols into free monomers, which subsequently provide more accessible substrates for the oxidases secreted by the *Bacillus*, thereby accelerating the overall transformation and consumption of phenolic compounds (39).

To gain deeper insights into the changes in polyphenolic composition, the contents of catechins monomers and gallic acid were quantified using HPLC (Table 1). Overall, all fermentation treatments led to a significant decrease in total catechins. Specifically, the total catechins content in the co-culture fermented infusion was 41.38% and 2.38% lower than that in the unfermented and *D. hansenii* mono-culture infusions, respectively. This decrease was primarily driven by the drastic conversion of ester catechins. In the co-culture infusion, the total content of ester catechins was substantially reduced by 92.63%, 21.20%, and 20.08% compared to the unfermented, *D. hansenii* mono-culture, and *B. subtilis* mono-culture infusions, respectively. This result may be attributed to the synergistically enhanced activities of polyphenol oxidase, tannase and esterase in the co-culture system: the former promotes the oxidative polymerization of ester catechins to form tea pigments (20), while the latter efficiently hydrolyzes their ester bonds, yielding corresponding non-ester catechins and gallic acid (40). *Bacillus* species, including *B. subtilis*, are well-documented producers of extracellular tannase (tannin acyl hydrolase), an enzyme that catalyzes the hydrolysis of ester bonds in ester catechins such as EGCG and ECG (41, 42). Recent studies have confirmed that *B. subtilis* strains isolated from fermented teas exhibit significant tannase activity, which contributes to the degradation of ester catechins and the consequent accumulation of gallic acid (41). This speculation is consistent with the observed significant accumulation of gallic acid in the co-culture fermented infusion in this study. In comparison, the total content of non-ester catechins in the co-culture infusion did not change significantly relative to the mono-culture treatments. This suggests that non-ester catechins are in a dynamic equilibrium during fermentation: they are generated as hydrolysis products of ester catechins, while simultaneously serving as precursors for oxidative polymerization reactions forming tea pigments or being directly metabolized and transformed by microorganisms (20).

**Table 1:** Effect of co-culture with *D. hanseni*i and *B. subtilis* on catechins and gallic acid in dark tea infusion.

Compounds	Contents (μg/mL)			
	NF	BSF	DHF	BDF
GA	31.51 ± 0.19a	2.80 ± 0.05c	3.08 ± 0.01c	12.00 ± 2.31b
GC	12.89 ± 0.08a	1.59 ± 0.17b	2.34 ± 0.96b	1.43 ± 0.39b
EGC	136.14 ± 1.15c	118.17 ± 0.01d	142.87 ± 0.46a	140.72 ± 0.07b
C	16.84 ± 2.70b	23.29 ± 0.16a	20.50 ± 0.35a	21.87 ± 0.54a
EC	2.96 ± 0.10b	3.45 ± 0.04a	1.67 ± 0.01c	1.67 ± 0.01c
EGCG	38.62 ± 0.22a	2.41 ± 0.23b	2.19 ± 0.19b	2.09 ± 0.06b
GCG	66.77 ± 1.07a	3.43 ± 0.16b	4.66 ± 0.12b	4.28 ± 0.74b
ECG	15.06 ± 2.09a	2.98 ± 0.20b	1.79 ± 0.42b	2.17 ± 0.12b
CG	9.72 ± 0.37a	3.18 ± 0.11b	3.52 ± 0.38b	1.04 ± 0.57c
Non-ester catechins	168.84 ± 2.02a	146.51 ± 0.20b	167.37 ± 0.63a	165.69 ± 0.45a
Ester catechins	130.16 ± 2.16a	12.00 ± 0.04b	12.17 ± 0.36b	9.59 ± 2.42b
Total catechins	299.00 ± 0.85a	158.51 ± 0.22c	179.54 ± 0.55b	175.27 ± 2.17b

Different letters (a-d) in the same line indicate significant difference (*p* < 0.05).

### 3.2 . Effects of co-culture fermentation on the profile of VOCs in dark tea infusion

#### 3.2.1 Changes in VOCs profile

**Figure 2:**
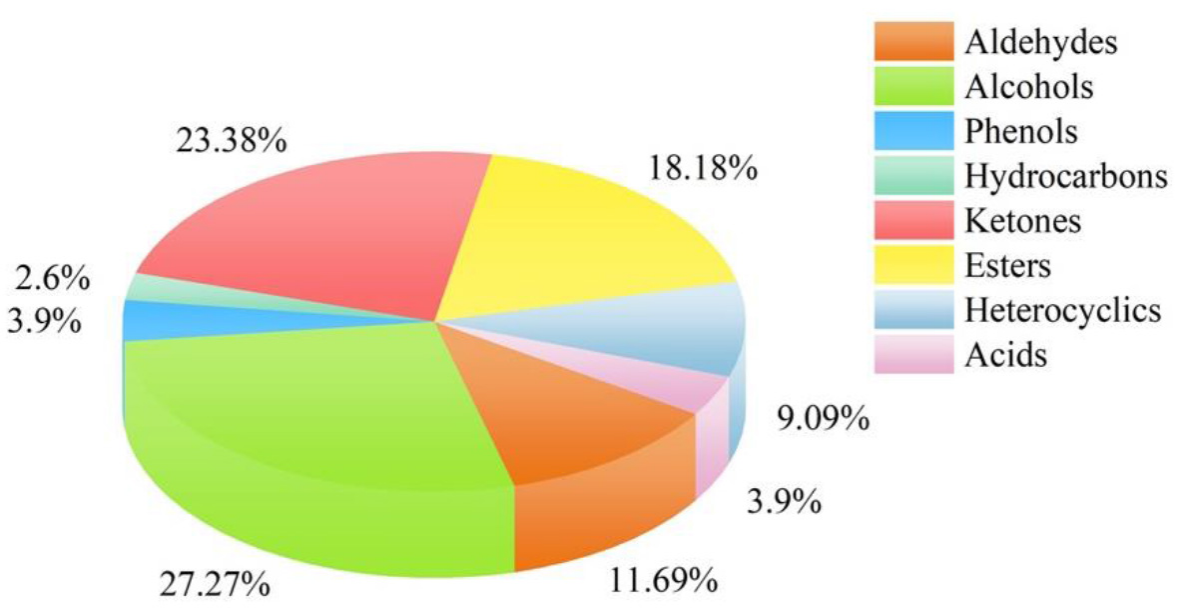
Classification of volatile organic compounds detected by GC-MS.

**Figure 3:**
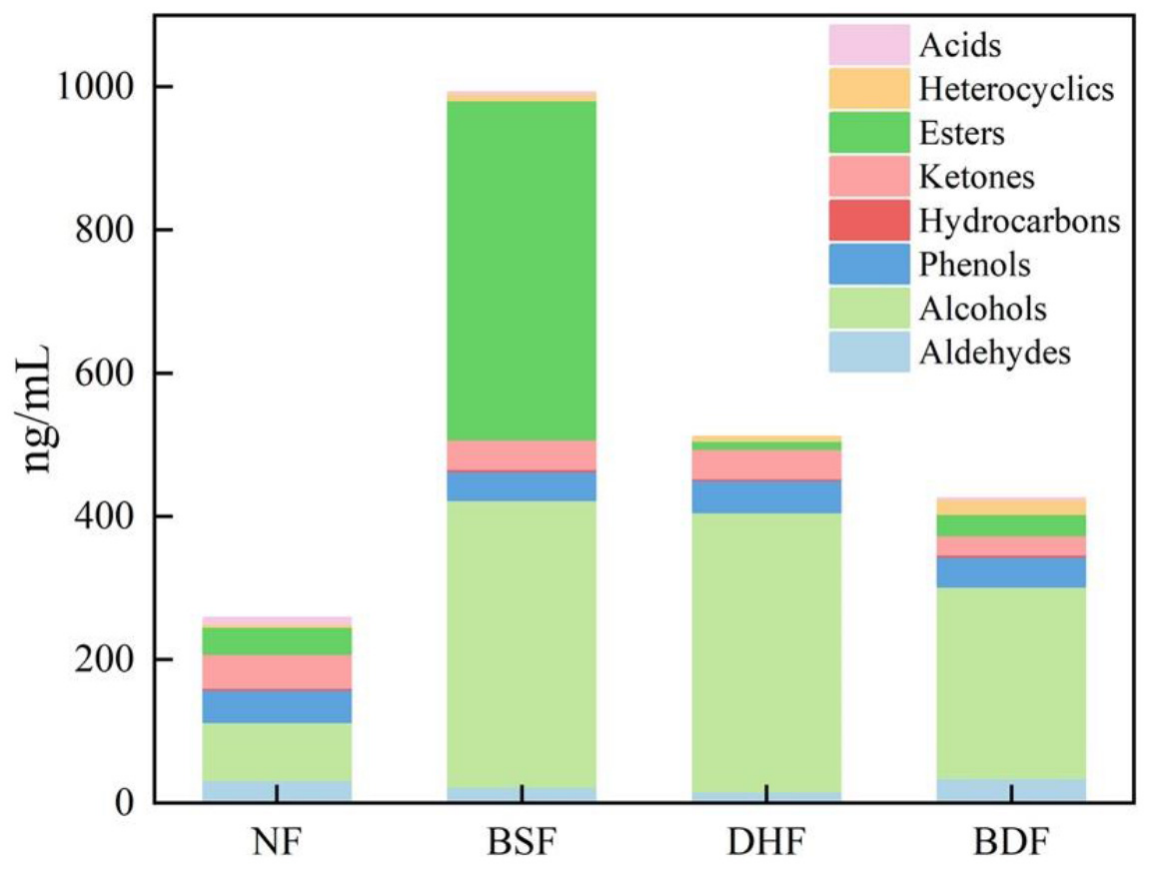
Influence of *D. hansenii* and *B. subtilis* co-culture on VOC profiles in dark tea infusion.

A total of 77 VOCs were identified from all samples by HS-SPME-GC-MS analysis, primarily including alcohols (21 compounds, 27.27%), ketones (18 compounds, 23.37%), esters (14 compounds, 18.18%), and aldehydes (7 compounds, 9.09%) (Figure 2). As shown in Figure 3, compared to the unfermented infusion, the total VOC content significantly increased in the *B. subtilis* mono-culture, *D. hansenii* mono-culture, and co-culture fermented infusions by 284.37%, 98.07%, and 64.67%, respectively. The marked increase in alcohols (average increase 337.64%) was the main driver for the rise in total VOCs. This result aligns with the microbial metabolic pathway where ketones are readily reduced to alcohols during fermentation (43).

**Figure 4:**
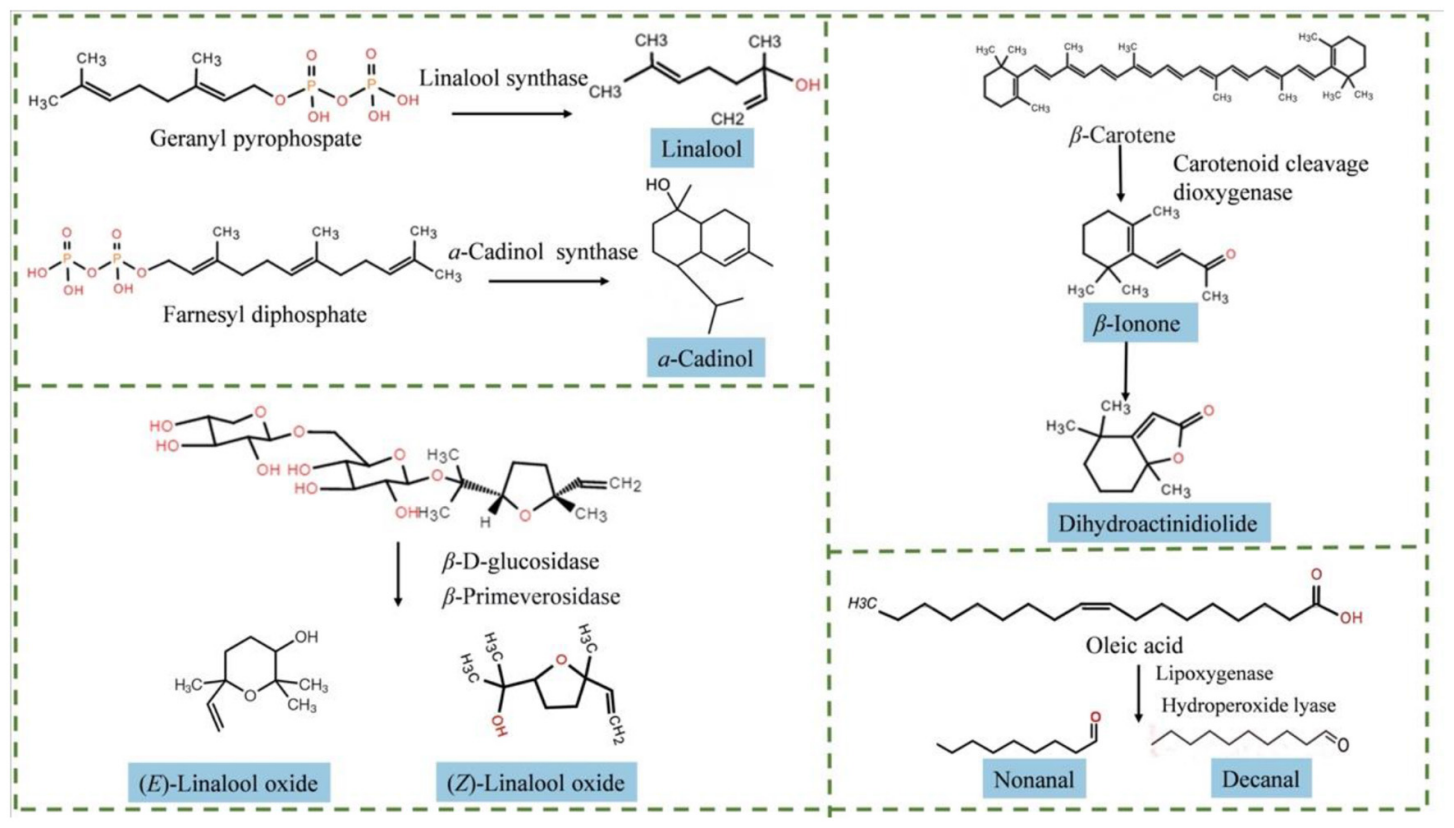
Possible metabolic pathways of key aroma compounds in co-culture infusion.

Table 2 revealed the uniqueness of the aroma composition in the co-culture fermented infusion: it retained some VOCs common to both mono-cultures while also forming its own distinct compositional profile. For instance, linalool, which imparts a characteristic lavender floral note, is a key component of the “fungal flower aroma” in Fu brick tea (44). The linalool content in the co-culture infusion significantly increased by 832.67% compared to the unfermented control, and its concentration was intermediate between those in the two mono-culture infusions. Its biosynthesis may originate from the catalysis of the geranyl pyrophosphate precursor by microbial linalool synthase (Figure 4) (45). Similarly, linalool oxides, presenting fresh woody, and floral notes, also accumulated significantly in the co-culture infusion. Notably, (*E*)-linalool oxide was newly formed during fermentation, and its content in the co-culture infusion increased by 7.85% and 163.84% compared to the two mono-culture infusions, respectively. This is attributed to the hydrolysis of glycosidic precursors by microbial enzymes like β-glucosidase or the direct conversion of linalool by P450 enzymes (Figure 4) (46–48). Furthermore, the contents of phenylethyl alcohol (rose-like aroma) and methyl salicylate (minty, fresh aroma) in the co-culture infusion were also intermediate between the two mono-cultures. These findings suggest that co-culture fermentation establishes a homeostatic regulation of key floral and woody aroma compounds, effectively balancing the excessive or insufficient aroma intensity often produced in mono-culture fermentation, thereby contributing to a more harmonious aromatic profile.

**Table 2:** Effect of co-culture with *D. hanseni*i and *B. subtilis* on VOCs in dark tea infusion.

No	Compounds	RI^a^/RI^b^	Identification^c^	CAS	Odor description	Content (ng/mL)			
						NF	BSF	DHF	BDF
Aldehydes									
a1	Octanal	1,294/1,289	MS, RI	124-13-0	Fat, soap, lemon, green	1.62 ± 0.62a	1.02 ± 0.16b	1.11 ± 0.49b	1.75 ± 0.37a
a2	Nonanal	1,396/1,391	MS, RI	124-19-6	Oily, floral	11.52 ± 1.24a	3.15 ± 0.61d	5.23 ± 0.51c	8.41 ± 0.73b
a3	Decanal	1,499/1,498	MS, RI	112-31-2	Soap, orange peel	2.65 ± 0.03c	4.03 ± 1.41b	4.47 ± 0.46b	12.62 ± 1.52a
a4	Benzaldehyde	1,524/1,520	MS, RI	100-52-7	Almonds	3.57 ± 0.29a	0.88 ± 0.16d	1.71 ± 0.20c	2.29 ± 0.17b
a5	β-Cyclocitral	1,615/1,611	MS, RI	432-25-7	Minty	ND	ND	3.52 ± 0.45b	4.35 ± 0.13a
a6	3,4-Dimethylbenzaldehyde	1,804/1,790	MS, RI	5973-71-7	-	9.98 ± 0.59a	9.99 ± 0.52a	ND	5.11 ± 0.37b
a7	Apricolin	2,020/2,024	MS, RI	104-61-0	-	1.73 ± 0.26b	3.94 ± 0.12a	ND	ND
a8	1-Ethyl-1H-pyrrole-2-carbaldehyde	1,607/1,610	MS, RI	2167-14-8	-	0.87 ± 0.38	ND	ND	ND
a9	Citral	1,729/1,718	MS, RI	5392-40-5	Lemon	0.35 ± 0.04	ND	ND	ND
Alcohols									
a10	(*Z*)-Linalool oxide	1,443/1,445	MS, RI	5989-33-3	Floral	3.82 ± 0.65d	68.32 ± 0.20c	105.56 ± 6.88a	84.42 ± 2.90b
a11	2-Ethy-1-hexanol	1,491/1,491	MS, RI	104-76-7	Earthy	0.46 ± 0.06c	2.54 ± 0.57b	10.48 ± 1.83a	11.18 ± 0.50a
a12	Linalool	1,550/1,547	MS, RI	78-70-6	Floral, sweet	13.99 ± 2.18c	114.37 ± 2.22b	229.61 ± 9.20a	130.48 ± 1.75b
a13	1-Nonanol	1,659/1,660	MS, RI	143-08-8	Fat, green	2.03 ± 0.19b	2.17 ± 0.25b	11.87 ± 3.93a	3.47 ± 0.62b
a14	α-Terpineol	1,694/1,697	MS, RI	98-55-5	Anise, mint	24.11 ± 3.96c	44.36 ± 0.56a	32.21 ± 2.45b	26.93 ± 0.84bc
a15	Nerol	1,798/1,797	MS, RI	106-25-2	Fresh, citrus, floral	3.39 ± 0.53b	13.00 ± 0.54a	ND	ND
a16	α-Santalol	1,836/1,843	MS, RI	115-71-9	Floral	0.33 ± 0.09b	0.39 ± 0.04a	ND	ND
a17	Geraniol	1,845/1,847	MS, RI	106-24-1	Floral, rose	20.75 ± 3.34a	0.20 ± 0.05b	ND	1.67 ± 0.08b
a18	Benzyl alcohol	1,874/1,870	MS, RI	100-51-6	Floral, sweet	ND	2.53 ± 0.35	ND	ND
a19	Phenethyl alcohol	1,907/1,906	MS, RI	60-12-8	Floral, rose	2.13 ± 0.04b	128.22 ± 0.28a	0.29 ± 0.05b	6.94 ± 0.61b
a20	β-Ionol	1,935/1,954	MS, RI	22029-76-1	Floral	1.38 ± 0.49b	2.23 ± 0.86a	ND	ND
a21	Nerolidol	2,038/2,033	MS, RI	7212-44-4	Floral, green, citrus	0.44 ± 0.15b	1.50 ± 0.24a	ND	ND
a22	Dehydrolinalool	1,612/1,613	MS, RI	29957-43-5	-	1.25 ± 0.14	ND	ND	ND
a23	(*Z*)-Sesquisabinene hydrate	2,073/2,082	MS, RI	58319-05-4	-	ND	0.35 ± 0.06a	ND	0.34 ± 0.13a
a24	Cedrol	2,101/2,116	MS, RI	77-53-2	Woody	1.65 ± 0.11b	2.15 ± 0.17a	ND	1.76 ± 0.10b
a25	Viridiflorol	2,086/2,095	MS, RI	552-02-3	Green, sweet	0.31 ± 0.10b	0.64 ± 0.04a	ND	ND
a26	1-Isopropyl-4-methyl-3-cyclohexen-1-ol	1,599/1,602	MS, RI	28219-82-1	-	ND	ND	1.36 ± 0.14b	4.46 ± 0.51a
a27	(*E*)-Linalool oxide	1,735/1,721	MS, RI	14049-11-7	Floral	ND	4.33 ± 0.32a	1.77 ± 0.29b	4.67 ± 0.19a
a28	1-Dodecanol	1,964/1,966	MS, RI	112-53-8	-	ND	ND	1.36 ± 0.39b	2.61 ± 0.43a
a29	*a*-Cadinol	2,189/2,187	MS, RI	19435-97-3	Herb, woody	ND	ND	ND	0.32 ± 0.09
a30	6-Methyl-5-hepten-2-ol	1,465/1,465	MS, RI	1569-60-4	-	ND	5.04 ± 0.81	ND	ND
Phenol									
a31	m-Cresol	2,175/2,187	MS, RI	123-07-9	-	1.51 ± 0.93b	32.59 ± 4.55a	0.23 ± 0.07b	23.12 ± 1.92a
a32	2,4-Di-tert-butyl-6-methylphenol	2,082/2,091	MS, RI	108-39-4	-	1.20 ± 0.03a	0.84 ± 0.09b	ND	ND
a33	2,4-Di-t-butylphenol	2,314/2,318	MS, RI	96-76-4	-	44.40 ± 5.59a	39.82 ± 2.14b	44.77 ± 4.26a	41.98 ± 3.12b
Hydrocarbon									
a34	Naphthalene	1,732/1,745	MS, RI	91-20-3	Woody	2.60 ± 0.13b	2.89 ± 0.55a	2.08 ± 0.14c	2.82 ± 0.07a
a35	3,4′-Diethyl-1,1′-biphenyl	2,227/2,228	MS, RI	61141-66-0	-	ND	ND	0.20 ± 0.04	ND
Ketones									
a36	6-Methylhept-5-en-2-one	1,341/1,338	MS, RI	110-93-0	Citrus, mushroom	3.77 ± 0.27a	1.96 ± 0.39b	3.48 ± 0.90a	4.64 ± 0.94a
a37	6-Methyl-3,5-heptadiene-2-one	1,591/1,602	MS, RI	1604-28-0	Citrus, grassy	3.31 ± 0.48a	2.45 ± 0.19b	ND	ND
a38	Acetophenone	1,648/1,647	MS, RI	98-86-2	Floral	3.13 ± 0.12b	9.43 ± 0.42a	ND	7.45 ± 0.40a
a39	2,6,6-Trimethyl-2-cyclohexene-1,4-dione	1,688/1,676	MS, RI	1125-21-9	Floral	1.07 ± 0.31c	ND	12.95 ± 1.02a	7.15 ± 0.25b
a40	β-Damascenone	1,813/1,801	MS, RI	23696-85-7	Fruity, sweet	2.91 ± 0.15a	2.28 ± 0.14a	1.03 ± 0.14b	0.66 ± 0.25c
a41	α-Ionone	1,842/1,840	MS, RI	127-41-3	Violet, woody, floral	ND	0.43 ± 0.11	ND	ND
a42	β-Ionone	1,930/1,940	MS, RI	79-77-6	Violet, woody, floral	11.37 ± 0.86a	12.39 ± 1.06a	0.58 ± 0.03b	0.72 ± 0.05b
a43	β-Ionone epoxide	1,981/1,962	MS, RI	23267-57-4	Fruity, sweet	5.34 ± 0.91b	7.37 ± 0.94a	ND	ND
a44	3,5-Octadien-2-one	1,520/1,522	MS, RI	38284-27-4	Sweet	9.53 ± 2.57	ND	ND	ND
a45	(*E, E*)-3,5-Octadien-2-one	1,569/1,570	MS, RI	30086-02-3	Grassy	6.13 ± 0.36a	3.77 ± 0.30b	0.27 ± 0.20c	0.47 ± 0.20c
a46	6,10-Dimethyl-5,9-undecadien-2-one	1,851/1,841	MS, RI	689-67-8	-	ND	ND	11.98 ± 2.90	ND
a47	Methyl nonyl ketone	1,597/1,593	MS, RI	112-12-9	-	ND	ND	3.74 ± 0.09	ND
a48	2,2,6-Trimethyl-1,4-cyclohexanedione	1,774/1,778	MS, RI	20547-99-3	-	ND	ND	1.47 ± 0.92	ND
a49	4-Hydroxyacetophenone	1,795/1,788	MS, RI	99-93-4	-	ND	ND	1.18 ± 0.05a	1.05 ± 0.07b
a50	Dihydro-β-ionone	1,825/1,842	MS, RI	17283-81-7	Violet, flower	ND	ND	0.74 ± 0.23b	1.39 ± 0.03a
a51	trans-β-Ionone	1,981/1,971	MS, RI	14901-07-6	Violet, woody, floral	ND	ND	3.56 ± 0.28a	3.14 ± 0.20a
a52	Benzophenone	2,465/2,450	MS, RI	119-61-9	Floral	0.43 ± 0.04a	0.38 ± 0.04b	0.46 ± 0.04a	0.23 ± 0.01c
15-7.4,-14.3690pta53	1-(4-Hydroxy-3-methylphenyl) ethanone	2,213/2,210	MS, RI	876-02-8	-	ND	0.74 ± 0.11	ND	ND
Esters									
a54	Ethyl safranate	1,976/1,981	MS, RI	35044-59-8	-	3.68 ± 0.40b	6.91 ± 0.60a	ND	ND
a55	Methyl salicylate	1,770/1,765	MS, RI	119-36-8	Mint, almond	14.54 ± 1.03b	443.28 ± 8.53a	4.59 ± 1.39c	14.03 ± 2.35b
a56	Ethyl laurate	1,840/1,841	MS, RI	106-33-2	Fresh leaf	ND	ND	1.11 ± 0.23a	1.17 ± 0.27a
a57	Ethyl tetradecanoate	2,044/2,049	MS, RI	124-06-1	Ether	0.33 ± 0.27c	1.64 ± 0.84c	3.47 ± 0.49b	7.16 ± 0.85a
a58	9-Hexadecenoic acid ethyl ester	2,267/2,281	MS, RI	54546-22-4	-	ND	ND	0.97 ± 0.16b	1.69 ± 0.86a
a59	(*E, E*)-farnesyl acetate	2,279/2,260	MS, RI	4128-17-0	Oil, wax	ND	ND	0.73 ± 0.31b	1.38 ± 0.09a
a60	Dihydroactinidiolide	2,321/2,331	MS, RI	17092-92-1	Woody	11.26 ± 0.70b	15.09 ± 0.48a	ND	1.86 ± 0.60c
a61	Eladic acid ethyl ester	2,454/2,476	MS, RI	6114-18-7	-	ND	ND	ND	0.27 ± 0.14
a62	Ethyl linoleate	2,521/2,521	MS, RI	544-35-4	Dairy	ND	ND	ND	0.90 ± 0.24
a63	Diisobutyl phthalate	2,534/2,536	MS, RI	84-69-5	-	6.57 ± 1.98a	4.14 ± 0.37b	0.74 ± 0.15c	1.22 ± 0.11c
a64	Ethyl hexanoate	1,239/1,233	MS, RI	123-66-0	Apple peel, fruity	0.64 ± 0.19	ND	ND	ND
a65	Ethyl caprylate	1,437/1,435	MS, RI	106-32-1	-	0.18 ± 0.05	ND	ND	ND
a66	Ethyl phenylacetate	1,784/1,783	MS, RI	101-97-3	Fruit, sweet	ND	0.88 ± 0.11	ND	ND
a67	(*Z*)-8-Dodecen-1-yl acetate	1,998/1,982	MS, RI	28079-04-1	-	1.04 ± 0.74b	1.60 ± 0.05a	ND	ND
Heterocyclics									
a68	Edulane	1,605/1,611	MS, RI	41678-29-9	Rose	1.15 ± 0.66b	4.12 ± 0.88a	ND	ND
a69	Coumaran	2,392/2,389	MS, RI	496-16-2	Green tea	ND	0.41 ± 0.02	ND	ND
a70	2-Propionylfuran	1,576/1,563	MS, RI	3194-15-8	-	ND	ND	1.92 ± 0.22a	0.67 ± 0.10b
a71	2,4,6-Trimethylpyridine	1,366/1,371	MS, RI	108-75-8	-	ND	ND	0.25 ± 0.01b	17.45 ± 8.22a
a72	1,2-Benzisothiazole	1,945/1,955	MS, RI	272-16-2	-	1.58 ± 0.14c	3.67 ± 0.12a	2.50 ± 0.16b	2.69 ± 0.28b
a73	2-Acetyl-1H-pyrrole	1,969/1,973	MS, RI	1072-83-9	-	ND	1.32 ± 0.34	ND	ND
a74	Indole	2,438/2,445	MS, RI	120-72-9	Mothball, burnt	1.33 ± 0.15a	0.58 ± 0.09b	0.16 ± 0.11c	ND
Acids									
a75	Octanoic acid	2,060/2,060	MS, RI	124-07-2	Cheese	3.00 ± 0.89a	0.33 ± 0.04b	ND	ND
a76	Nonanoic acid	2,166/2,171	MS, RI	112-05-0	Green, fat	4.18 ± 0.97a	ND	ND	0.62 ± 0.17b
a77	Decanoic acid	2,273/2,276	MS, RI	334-48-5	Dust, fat, grass	0.51 ± 0.17a	0.15 ± 0.03b	ND	ND

^a^, the retention index of the compound on HP-5MS;

^b^, the retention index of the compound in the reference;

^c^, MS, mass spectrum comparison using NIST17 library. RI, retention index in agreement with literature value;

ND, No data was detected in the sample. -: no odor description information was found in the literature. Different letters (a-d) in the same line indicate significant difference (*p* < 0.05).

Further analysis revealed that the co-culture fermented infusion integrated the aroma characteristics of both mono-cultures. It shared 12 VOCs with the *D. hansenii* mono-culture infusion, including dihydro-β-ionone (woody), trans-β-ionone (violet floral), ethyl laurate (fruity-sweet), and 2, 4, 6-trimethylpyridine (roasted nutty), among others. Among these, β-ionone may be produced via the oxidation of β-carotene by microbial dioxygenases (Figure 4) (49, 50). Concurrently, the co-culture infusion shared 6 VOCs with the *B. subtilis* mono-culture infusion, such as geraniol, acetophenone, and dihydroactinidiolide. Dihydroactinidiolide can be generated from the further oxidation of β-ionone (51). As a result, this integration of the distinct aroma profiles enabled the co-culture fermentation to overcome the inherent simplicity of its mono-culture counterparts, thereby establishing a more complex and well-rounded composite aroma.

Particularly noteworthy is the production of three unique VOCs exclusively identified in the co-culture fermented infusion: α-cadinol, eladic acid ethyl ester, and ethyl linoleate, suggesting cross-feeding and co-metabolic interactions. This agrees with Gao et al. (52), by incorporating multiple yeast and bacterial species into a synthetic microbiota, the co-culture system can generate a broader range of flavor compounds than mono-cultures. These results show microbial co-culture can activate hidden metabolic pathways and generate new metabolites via cross-feeding. Among these, α-cadinol—a sesquiterpene alcohol—imparts a soft and dry woody note to the aroma profile. Its synthesis involves the methylerythritol phosphate (MEP) and mevalonic acid (MVA) pathways and is catalyzed from farnesyl pyrophosphate by specific synthases (Figure 4) (15, 53). The two ethyl esters contribute oily and waxy notes. Additionally, the contents of nonanal and decanal, which impart citrus peel and fruity aromas, were also significantly elevated in the co-culture infusion. These aldehydes may originate from the oxidative cleavage of lipids (e.g., oleic acid) via the microbial lipoxygenase pathway (Figure 4) (45, 54). In summary, co-culture fermentation shaped a complex aroma profile, characterized by distinct floral, fruity, woody, and fatty notes, through the integration and enrichment of diverse VOCs.

#### 3.2.2 Identification of key aroma-active compounds based on relative odor activity values and multivariate statistical analysis

**Figure 5:**
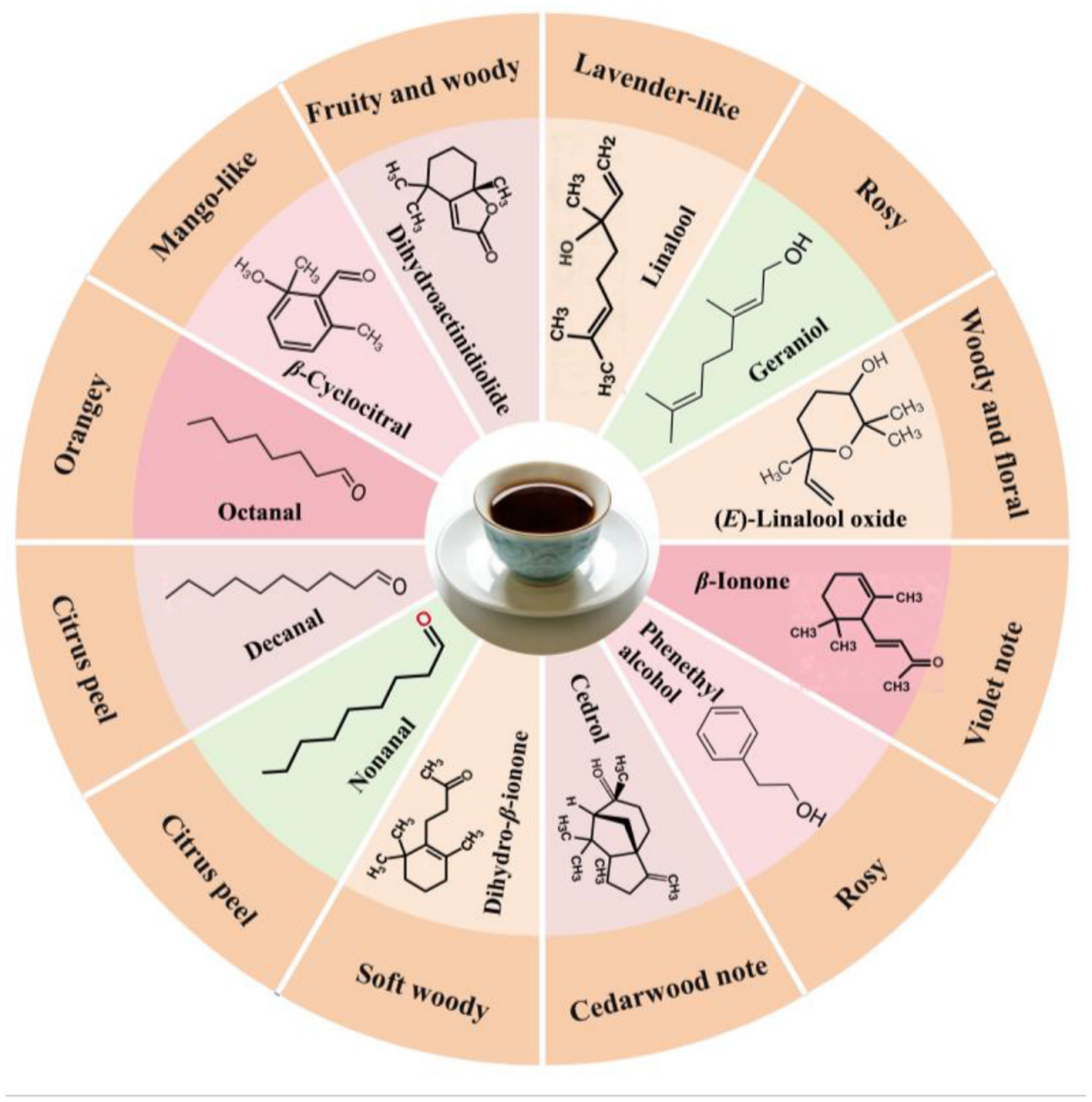
An aroma wheel diagram for the co-culture fermented dark tea infusion was constructed based on VOCs with rOAV > 1.

To assess the contribution of VOCs to the actual aroma, the relative odor activity value (rOAV) was calculated. It is well established that compounds with a rOAV greater than 1 are defined as active aroma compounds, which play a critical role in shaping the distinctive aroma profile (31, 32). As shown in Table 3, in the co-culture fermented infusion, 12 VOCs with rOAV > 1 were identified as key aroma-active compounds. Based on this, an aroma wheel for the co-culture infusion was constructed (Figure 5). Analysis indicated that nonanal, decanal, octanal, and β-cyclocitral primarily contributed fruity and fatty notes; linalool, geraniol, phenylethyl alcohol, (*E*)-linalool oxide, and β-ionone constituted the main floral body; while cedrol, dihydroactinidiolide, and dihydro-β-ionone imparted woody characteristics. The distinctive “fungal flower aroma” in Fu brick tea arises from the specific proportional blending of these aromatic components (44). Consequently, co-culture fermentation effectively shaped this complex aroma profile through the selective enrichment of corresponding key compounds.

**Table 3:** Impact of *D. hansenii* and *B. subtilis* co-culture on the rOAV of main VOCs in dark tea infusion.

Compounds	Thresholds (ng/mL)	rOAV			
		NF	BSF	DHF	BDF
Aldehydes					
Octanal	0.7	2.32	1.45	1.59	2.49
Nonanal	1	11.52	3.15	5.23	8.41
Decanal	3	0.88	1.34	1.49	4.21
Benzaldehyde	3	1.19	0.29	0.57	0.76
β-Cyclocitral	3	ND	ND	1.17	1.45
Citral	0.04	8.75	ND	ND	ND
Alcohols					
(*Z*)-Linalool oxide	320	0.01	0.21	0.33	0.26
Linalool	0.22	63.60	519.86	1,043.70	593.07
1-Nonanol	50	0.04	0.04	0.24	0.07
α-Terpineol	330	0.07	0.13	0.10	0.08
Geraniol	0.08	259.40	2.50	ND	20.85
Benzyl alcohol	100	ND	0.03	ND	ND
Phenethyl alcohol	0.02	106.62	6,411.08	14.60	346.93
Nerolidol	10	0.04	0.15	ND	ND
Cedrol	0.5	3.30	4.30	ND	3.52
(*E*)-Linalool oxide	2	ND	2.17	0.89	2.34
Phenol					
2,4-Di-t-butylphenol	500	0.09	0.08	0.09	0.08
Hydrocarbon					
Naphthalene	6	0.43	0.48	0.35	0.47
Ketones					
6-Methylhept-5-en-2-one	50	0.08	0.04	0.07	0.09
6-Methyl-3,5-heptadiene-2-one	100	0.03	0.02	ND	ND
Acetophenone	65	0.05	0.15	ND	0.11
β-Damascenone	10	0.29	0.23	0.10	0.07
α-Ionone	0.4	ND	1.08	ND	ND
β-Ionone	0.2	56.84	61.96	2.92	3.59
3,5-Octadien-2-one	0.15	63.53	ND	ND	ND
Dihydro-β-ionone	0.1	ND	ND	7.40	13.95
Esters					
Methyl salicylate	40	0.36	11.08	0.11	0.35
Ethyl tetradecanoate	500	< 0.01	< 0.01	0.01	0.01
Ethyl tetradecanoate	1.8	6.25	8.38	ND	1.04
Ethyl hexanoate	14	0.05	ND	ND	ND
Ethyl caprylate	13	0.01	ND	ND	ND
Ethyl phenylacetate	100	ND	0.01	ND	ND
Heterocyclics					
Indole	140	0.01	< 0.01	< 0.01	ND
Acids					
Octanoic acid	500	0.01	< 0.01	ND	ND
Nonanoic acid	3,559	< 0.01	ND	ND	< 0.01
Decanoic acid	7,200	< 0.01	< 0.01	ND	ND

ND, data was not detected in the sample.

**Figure 6:**
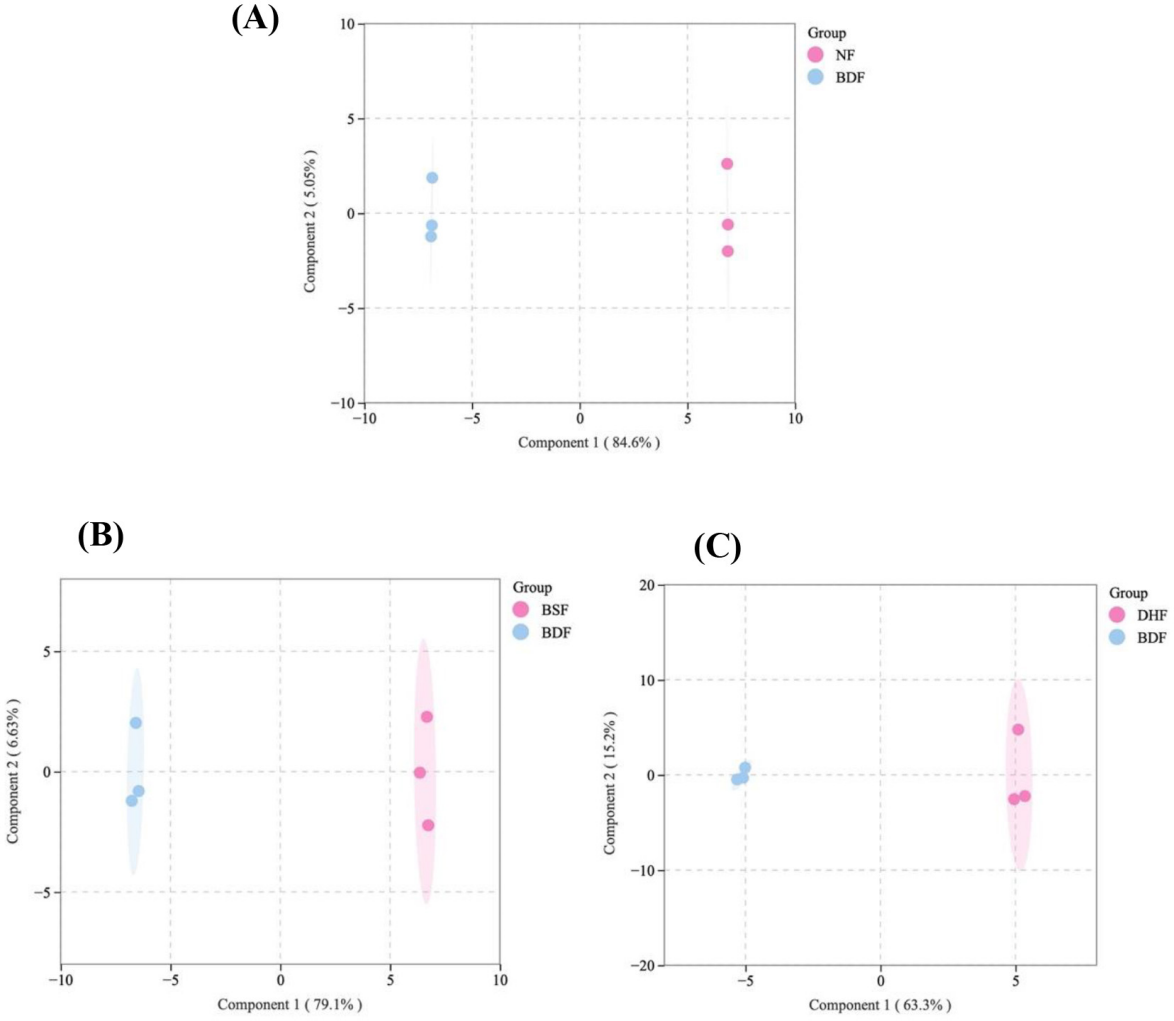
The score plots of OPLS-DA pairwise comparisons of GC-MS results **(A)** BDF vs.NF; **(B)** BDF vs. BSF; **(C)** BDF vs. DHF.

**Figure 7:**
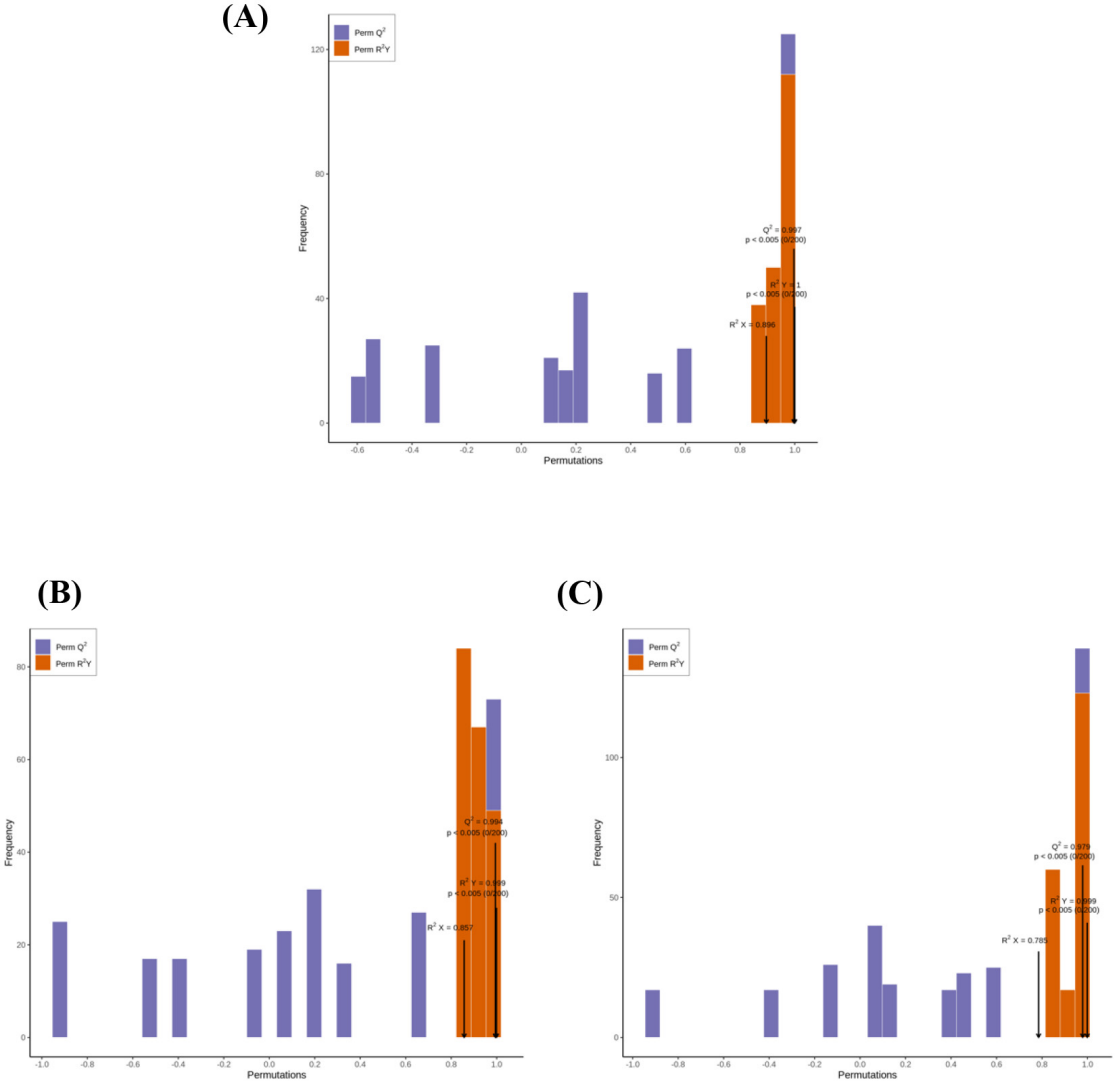
The permutation analysis of OPLS-DA pairwise comparison of GC-MS results. **(A)** BDF vs.NF; **(B)** BDF vs. BSF; **(C)** BDF vs. DHF.

**Figure 8:**
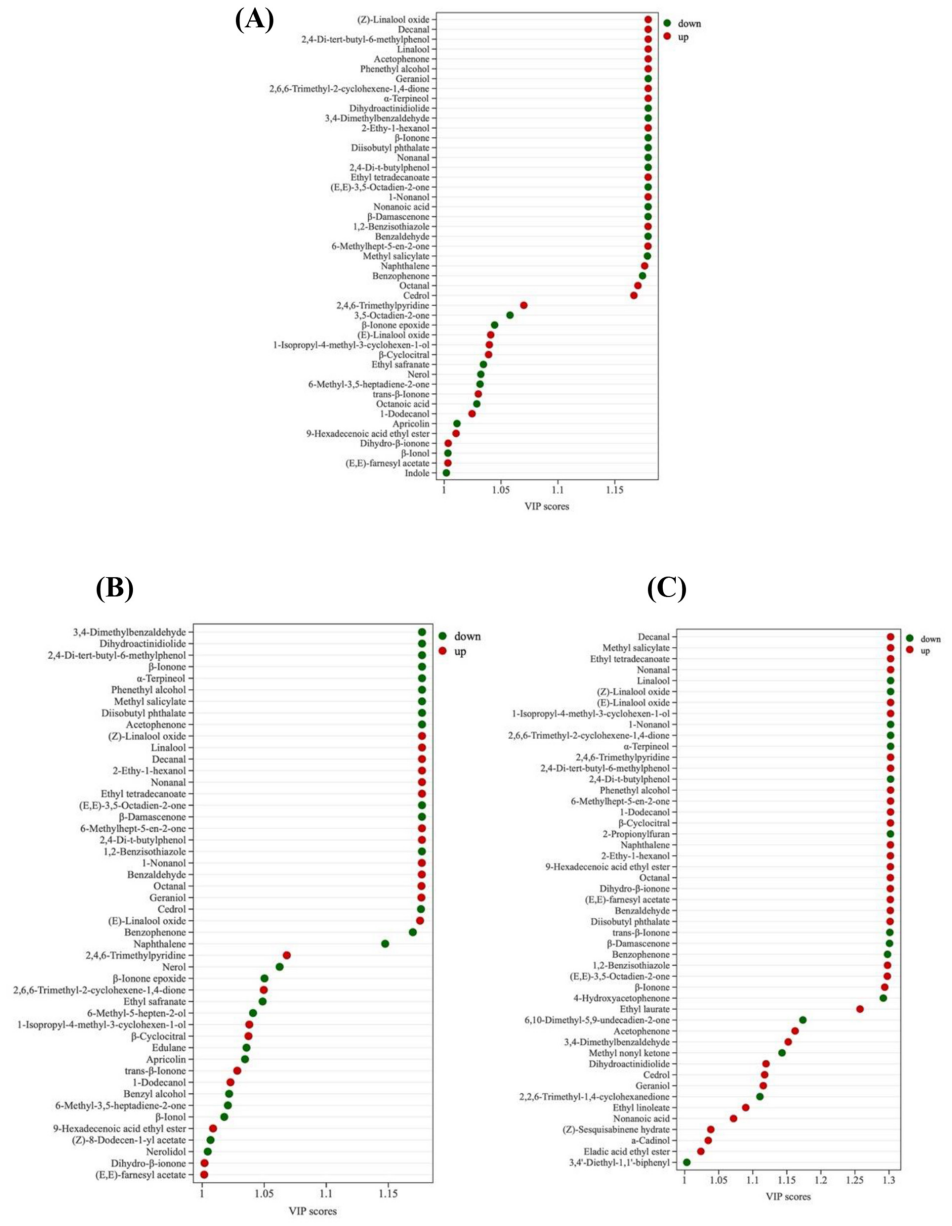
The VIP is derived from the OPLS-DA pairwise comparisons of GC-MS results. Red and green indicate higher and lower VOC concentrations, respectively. **(A)** BDF vs.NF; **(B)** BDF vs. BSF; **(C)** BDF vs. DHF.

Orthogonal Partial Least Squares Discriminant Analysis (OPLS-DA) models were developed to compare BDF (co-culture fermentation) with NF (unfermented tea), BSF (*B. subtilis* mono-culture), and DHF (*D. hansenii* mono-culture), in order to identify differentially expressed VOCs resulting from co-culture fermentation (Figure 6). The models revealed distinct separation between BDF and all other samples, indicating significant differences in their VOC profiles. Model robustness and validity were assessed via permutation tests, with R2Y, R2X, and Q2 values exceeding 0.5 (*p* < 0.05), confirming the reliability of the OPLS-DA models (Figure 7). Differential VOCs were identified according to the criteria of variable importance in projection (VIP) ≥ 1 and *p* < 0.05 (Figure 8). Comparative analysis demonstrated that, relative to BSF, BDF exhibited upregulation of 22 VOCs and downregulation of 26 VOCs. Similarly, compared to DHF, 34 VOCs were upregulated and 15 were downregulated in BDF. Furthermore, when compared with NF, BDF showed a significant alteration in VOC composition, with 25 VOCs markedly increased and 22 decreased. These substantial shifts in VOC expression provide direct evidence that co-culture fermentation profoundly remodels the volatile profile of tea infusion, thereby establishing the compositional foundation for its distinctive aroma properties.

### 3.3 Molecular docking analysis of key aroma compounds with olfactory receptors

To elucidate the role of key aroma compounds at the sensory perception level, molecular docking was conducted using the three most abundant VOCs identified by rOAV in the co-culture infusion—linalool, geraniol, and phenylethyl alcohol—with established olfactory receptors known to recognize aromatic alcohols: Olfactory receptor family 1 subfamily A member 1 (OR1A1), Olfactory receptor family 1 subfamily G member 1 (OR1G1), and Olfactory receptor family 2 subfamily W member 1 (OR2W1) (55, 56). These receptors—OR1A1, OR1G1, and OR2W1—were selected based on their broad ligand specificity and well-documented involvement in the perception of aromatic alcohol-type tea volatiles, such as linalool, geraniol, and phenylethyl alcohol, which contribute significantly to the floral aroma characteristics of tea (55, 56). In our study, these key volatile compounds exhibited high rOAV in the co-culture fermented dark tea infusion, underscoring their sensory relevance. Therefore, this molecular docking approach offers a mechanistic perspective on how these key VOCs may enhance the floral aroma profile of co-culture fermented dark tea via specific interactions with these olfactory receptors.

**Figure 9:**
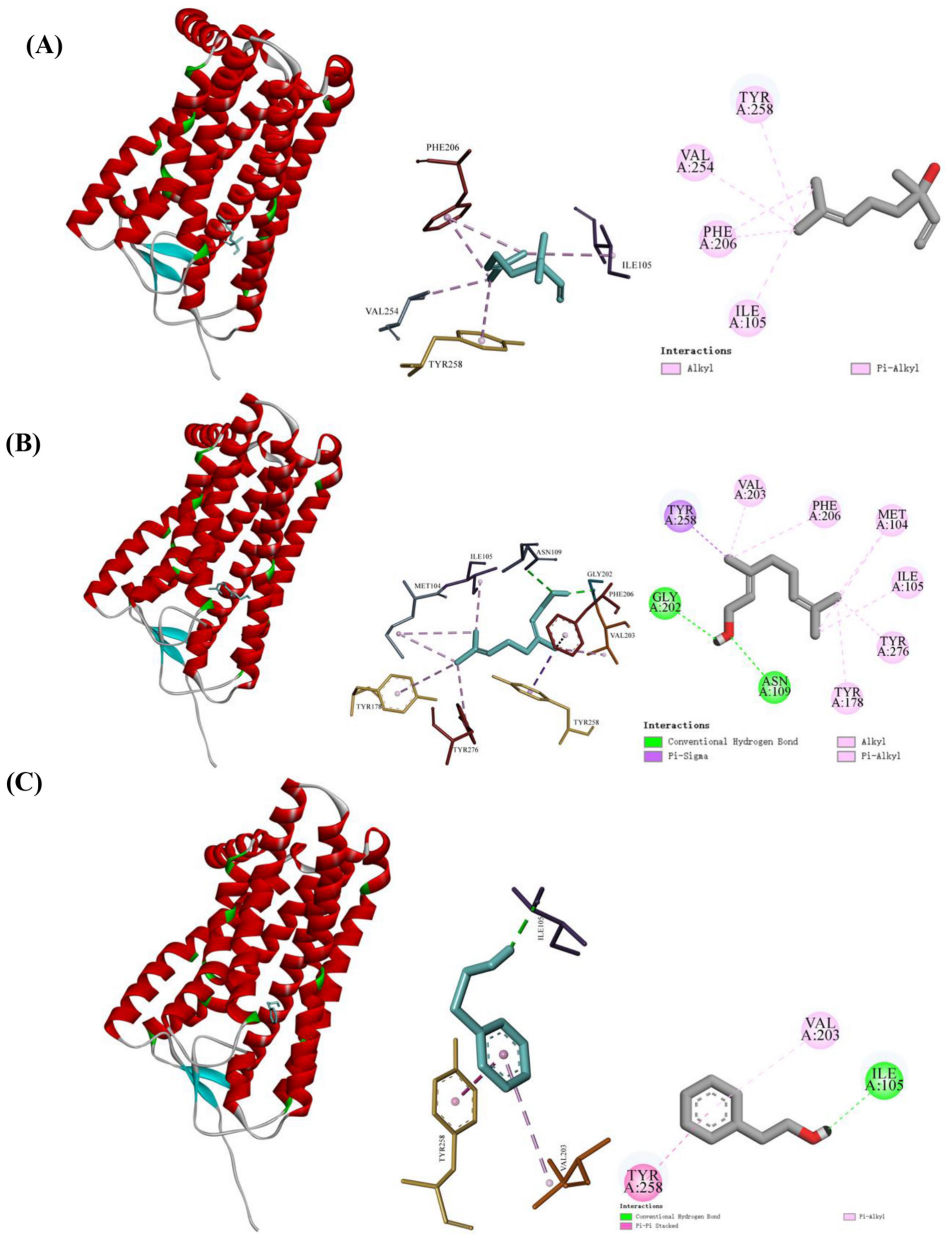
Molecular docking results of three key floral VOCs with olfactory receptor OR1A1 **(A)** linalool; **(B)** geraniol; **(C)** phenylethyl alcohol.

Analysis of the molecular docking results (Table 4) revealed that the mean binding energies of linalool, geraniol, and phenylethyl alcohol toward the three olfactory receptors were −6.27, −5.93, and −5.73 kcal/mol, respectively. Lower binding energy reflects stronger affinity (56); accordingly, linalool displayed the strongest binding, whereas phenylethyl alcohol exhibited the weakest. Linalool's tertiary alcohol group and cyclic alkene structure enable extensive hydrophobic interactions with multiple residues in the olfactory receptor binding pocket, stabilizing the complex more effectively than the linear chain structures of geraniol and phenylethyl alcohol (57). The relatively lowest binding energy of linalool among the three compounds is consistent with its highest rOAV (593.07), indicating a correlation between its predicted receptor interaction and its high odor activity. All three ligands showed the strongest binding toward OR1A1. This observation may be attributed to OR1A1's role as a broad-spectrum olfactory receptor that also exhibits specific recognition capability toward certain monoterpenes, such as linalool (58). Visualization of the docking poses (Figure 9) illustrates the three-dimensional and two-dimensional interaction profiles. As shown, linalool engages in hydrophobic contacts with Ile 105, Phe 206, Val 254, and Tyr 258 of OR1A1, consistent with earlier reports (55). The high binding affinity of linalool for OR1A1 likely arises from these extensive hydrophobic interactions with multiple residues, which substantially stabilize the receptor–ligand complex (59). Geraniol forms hydrogen bonds with Gly 202 and Asn 109 of OR1A1 and participates in hydrophobic interactions with Tyr 258, Val 203, Phe 206, Met 104, Ile 105, Tyr 276, and Tyr 178. Similarly, phenylethyl alcohol displays hydrophobic contacts with Val 203 and Tyr 258 while establishing a hydrogen bond with Ile 105 of OR1A1. In summary, the molecular docking data indicate that OR1A1 is likely a core molecular target involved in the perception of these key floral aroma compounds in the co-culture fermented tea infusion.

**Table 4:** Summary of the binding energy between three floral aroma key VOCs and olfactory receptors.

Ligands	Binds energy (kcal/mol)		
	Linalool	Geraniol	Phenylethyl alcohol
OR1A1	−7.40	−6.80	−6.30
OR1G1	−5.00	−5.40	−5.50
OR2W1	−6.40	−5.60	−5.40
Average	−6.27	−5.93	−5.73

OR1A1, Olfactory receptor family 1 subfamily A member 1; OR1G1, Olfactory receptor family 1 subfamily G member 1; OR2W1, Olfactory receptor family 2 subfamily W member 1.

### 3.4 Sensory evaluation

**Figure 10:**
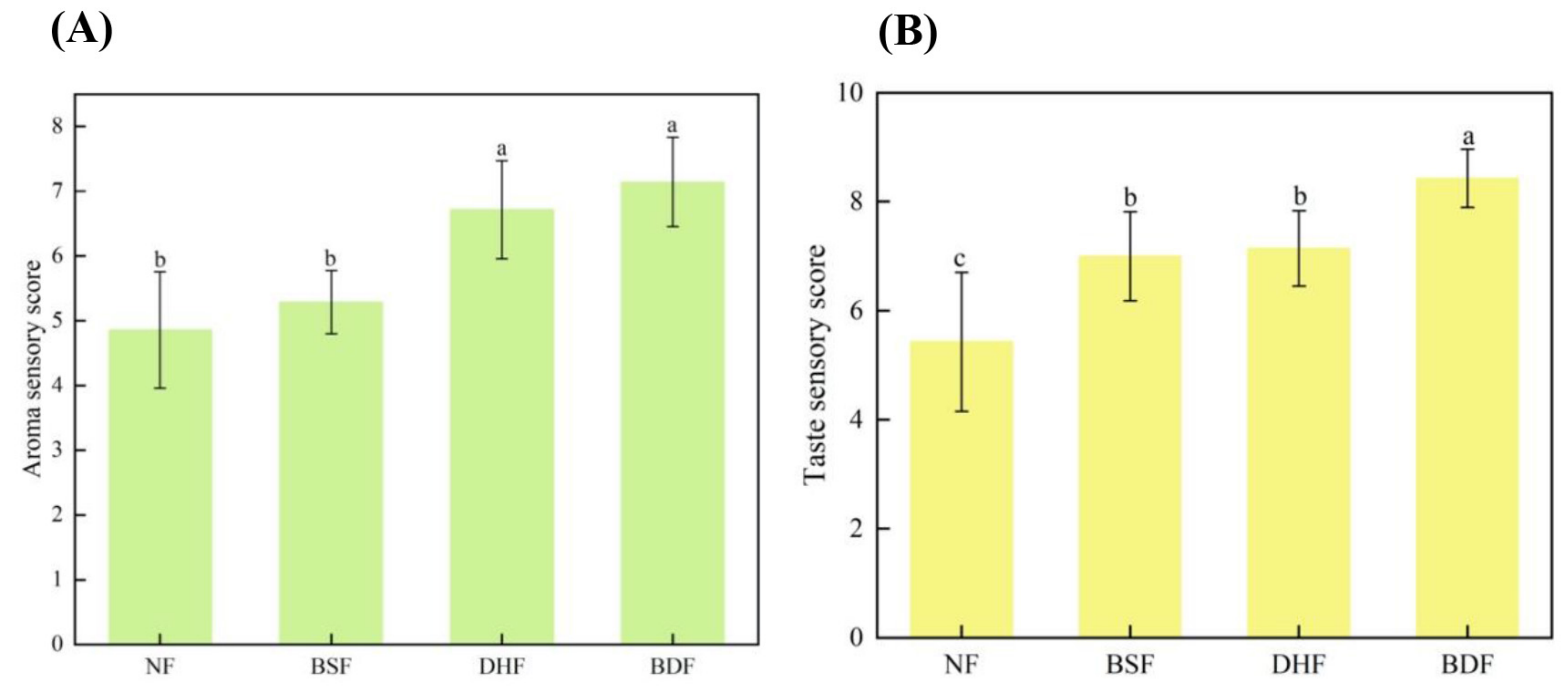
Effect of co-culture fermentation with *D. hansenii* and *B. subtilis* on the sensory profile of dark tea infusion **(A)** aroma sensory score; **(B)** taste sensory score. Means followed by different letters indicate statistically significant differences (*p* < 0.05) among the samples.

**Figure 11:**
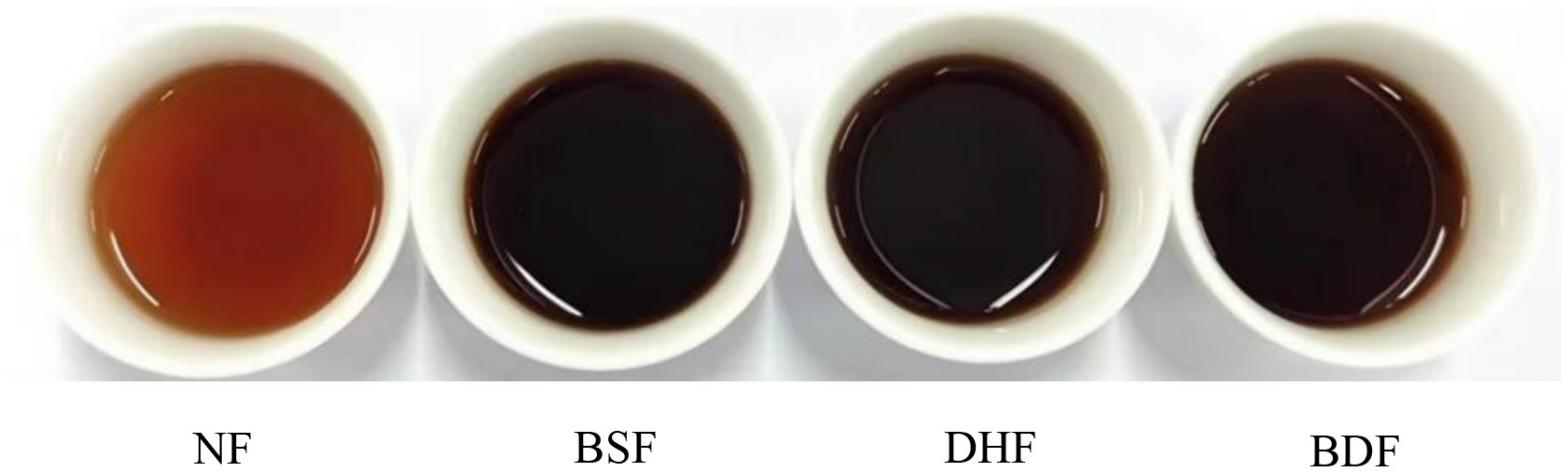
*D. hansenii* and *B. subtilis* co-culture fermentation modulates the color of dark tea infusion.

Sensory evaluation results (Figure 10) comprehensively confirmed the quality improvement achieved through co-culture fermentation. The infusion fermented by co-culture received the highest overall acceptability score, demonstrating a particularly pronounced advantage in taste (score 8.43) over all other treatments. Regarding taste profile, while both mono-culture infusions exhibited certain mellowness, they retained perceptible bitterness and astringency. In contrast, the co-culture infusion showed significantly enhanced mellowness and a lingering sweet aftertaste, alongside substantially reduced bitterness and astringency, leading to superior overall palatability. In terms of aroma, scores for *D. hansenii* monoculture and co-culture fermented infusions were significantly higher than those of the unfermented control and *B. subtilis* monoculture. Specifically, the *B. subtilis* monoculture infusion developed undesirable off-odors, whereas the *D. hansenii* monoculture infusion presented a relatively pure aroma, albeit with a faint green note. The co-culture infusion, however, exhibited a pure, fresh, and well-balanced aromatic profile without any off-notes. These findings suggest that *D. hansenii* plays a key role in aroma enhancement, and its co-fermentation with *B. subtilis* generates a positive synergistic effect that optimizes aromatic quality and complexity. Additionally, all fermentation treatments deepened the infusion color from reddish-brown to dark brown (Figure 11). Previous studies have shown that theabrownins are high-molecular-weight compounds formed from substances such as catechins through reactions including condensation, polymerization, oxidation, and coupling, and that an increase in their content darkens the color of tea infusions (8, 60, 61). In this study, *D. hansenii* and *B. subtilis* may have produced enzymes such as peroxidase, polyphenol oxidase, and laccase during fermentation, promoting the conversion of catechins into theabrownins, which resulted in a darker color of the tea infusion after co-fermentation (60, 61). In summary, co-culture fermentation of *D. hansenii* and *B. subtilis* synergistically improves the taste and aroma profiles of dark tea infusions, leading to a significant enhancement in the overall sensory quality of the dark tea beverage.

## 4 Conclusion

This study systematically investigated the effects of mono-culture fermentation by *B. subtilis* and *D. hansenii*, as well as their co-fermentation, on the flavor quality of dark tea infusion. The results demonstrated that co-culture fermentation significantly modulates the composition of key flavor-related compounds through synergistic interactions. Specifically, co-fermentation markedly reduced the total polyphenols content (decreasing by 45.12%, 38.96%, and 54.14% compared to non-fermented, *B. subtilis* single-fermented, and *D. hansenii* single-fermented infusions, respectively) and the level of ester catechins (decreased by 92.63% compared to the non-fermented infusion), effectively diminishing the sources of astringency and bitterness. Concurrently, co-culture fermentation optimized the proportion and diversity of aroma compounds, generating unique constituents such as α-cadinol, eladic acid ethyl ester, and ethyl linoleate, while retaining the common aromatic attributes present in both mono-culture fermentation treatments. This resulted in a well-balanced and multi-layered aromatic profile dominated by floral, fruity, and woody notes. OPLS-DA confirmed that the volatile composition of the co-culture fermented infusion was distinct from those of mono-culture fermented and non-fermented infusions. The rOAV analysis further identified that aldehydes, terpenic alcohols, and lactones primarily contributed to fruity, floral, and woody aroma characteristics, respectively. Molecular docking results indicated that key floral aroma compounds, such as linalool, exhibited strong binding affinity to the olfactory receptor OR1A1. Sensory evaluation consistently demonstrated that the co-culture fermented infusion received the highest overall scores in taste and aroma. It was characterized by a mellow and sweet aftertaste with reduced bitterness and astringency, alongside a pure, refreshing, and well-balanced fragrance. In summary, the co-fermentation of *D. hansenii* and *B. subtilis* effectively improved the flavor characteristic of dark tea infusion by synergistically regulating the transformation of polyphenols and the composition of VOCs. These findings provide new insights into the evolution of flavor substances and phenolic metabolites during the mixed fermentation of dark tea infusion by *D. hansenii* and *B. subtilis*. and a technical basis for the development of flavor-oriented fermented tea beverages. Future studies could focus on optimizing the inoculation ratio and fermentation parameters for industrial scale-up, elucidating the precise molecular pathways of the microbial cross-talk through multi-omics analysis, and evaluating the health benefits of the co-fermented tea beverage.

## Data Availability

The original contributions presented in the study are included in the article/supplementary material, further inquiries can be directed to the corresponding authors.
